# Case of Trismus and Spasms of the Whole Body, Arising from Hysteria

**Published:** 1818-07

**Authors:** John Maclean

**Affiliations:** Edinburgh.


*For the London Medical and Physical Journal.*

### *Case of Trismus and Spasms of the whole Body, arising from Hysteria;* by John Maclean, M.D. Edinburgh.

JANE FORSYTH, aged 15, of a very robust frame and sanguine temperament, having never menstruated, was suddenly seized with violent hysteric affections, attended with a degree of mental derangement, exciting her to leap upon tables and chairs, to the great alarm of the ladies in whose family she lived.

These symptoms had continued some time when I first saw the patient: I ordered her to be secured by the servants, got her to use the pediluvium, and bled her at the ankle.

This c mposed her for the night, but, for several days afterwards, she was affected with alternate fits of laughing and crying, and strong spasms of the limbs, extending to the body, which was ultimately so rigid as to render it extremely difficult to move her.

Her jaws subsequently became firmly closed, with little intermission for two days. I directed laxative glysters to be administered, which operated slightly; a large opiate plaster to the neck; and, in the intervals of relaxation, pills of camphor and musk; but without any very sensible benefit. The only remedies from which I could perceive any alleviation to the trismus and general spasms were the semicupium and repeated bleedings at the ankles, which, at the same time, apparently induced a slight degree of menstruation: whereupon, judging the whole of the complaint to depend on the deficiency of this discharge, I ordered her the tinct. hellebori nigri, in full doses every night and morning, and the semicupium every night. Under this treatment, a copious menstruation took place; the spasmodic symptoms subsided; and, in a few weeks, she got perfectly well. For a considerable time afterwards, however, she was subject to hysteric paroxysms at every monthly period, but never had any return of the locked jaw.

I have communicated this case the more readily, as, upon my mentioning it to Dr. Gregory, he seemed to think it remarkable, and said it was one of the few cases he had known of locked jaw occurring from hysteria.

The occasional efficacy of the tinct. hellebori nigri as an emmenagoque, was strikingly displayed in another case which occurred to me.

A young lady had been in a state of derangement, from agitation of mind, for some time, and during several months had entirely ceased to menstruate. I ordered the tinct. hellebori nigri to be administered in the usual doses; but, the phial containing it happening to be left in her apartment, when alone, she took spontaneously the amount of two or three doses at once. The next day, her menstruation was restored, she almost instantly recovered her mental faculties, and has continued well ever since.

*Edinburgh; May* 15, 1818.

